# Evaluation of pressure-induced pain in patients with disorders of consciousness based on functional near infrared spectroscopy

**DOI:** 10.3389/fneur.2025.1542691

**Published:** 2025-04-07

**Authors:** Tan Zhang, Nan Wang, Xiaoke Chai, Qiheng He, Tianqing Cao, Liqun Yuan, Qing Lan, Yi Yang, Jizong Zhao

**Affiliations:** ^1^Department of Neurosurgery, The Second Affiliated Hospital of Soochow University, Suzhou, China; ^2^Department of Neurosurgery, Beijing Tiantan Hospital, Capital Medical University, Beijing, China; ^3^China National Clinical Research Center for Neurological Diseases, Beijing, China; ^4^Department of Neurosurgery, Peking Union Medical College Hospital, Chinese Academy of Medical Sciences and Peking Union Medical College, Beijing, China; ^5^Brain Computer Interface Transitional Research Center, Beijing Tiantan Hospital, Capital Medical University, Beijing, China

**Keywords:** disorders of consciousness, functional connectivity, hemodynamic responses, near-infrared spectroscopy, pressure-induced pain

## Abstract

**Objective:**

This study aimed to investigate the brain's hemodynamic responses (HRO) and functional connectivity in patients with disorders of consciousness (DoC) in response to acute pressure pain stimulation using near-infrared spectroscopy (NIRS).

**Methods:**

Patients diagnosed with DoC underwent pressure stimulation while brain activity was measured using NIRS. Changes in oxygenated hemoglobin (HbO) and deoxygenated hemoglobin (HbR) concentrations were monitored across several regions of interest (ROIs), including the primary somatosensory cortex (PSC), primary motor cortex (PMC), dorsolateral prefrontal cortex (dPFC), somatosensory association cortex (SAC), temporal gyrus (TG), and frontopolar area (FPA). Functional connectivity was assessed during pre-stimulation, stimulation, and post-stimulation phases.

**Results:**

No significant changes in HbO or HbR concentrations were observed during the stimulation vs. baseline or stimulation vs. post-stimulation comparisons, indicating minimal activation of the targeted brain regions in response to the pressure stimulus. However, functional connectivity between key regions, particularly the PSC, PMC, and dPFC, showed significant enhancement during the stimulation phase (*r* > 0.9, *p* < 0.001), suggesting greater coordination among sensory, motor, and cognitive regions. These changes in connectivity were not accompanied by significant activation in pain-related brain areas.

**Conclusion:**

Although pain-induced brain activation was minimal in patients with DoC, enhanced functional connectivity during pain stimulation suggests that the brain continues to process pain information through coordinated activity between regions. The findings highlight the importance of assessing functional connectivity as a potential method for evaluating pain processing in patients with DoC.

## 1 Introduction

Disorders of consciousness (DoC) refer to a spectrum of neurological conditions characterized by impaired consciousness and a lack of awareness of the external environment, including the Unresponsive Wakefulness Syndrome (UWS) and minimally conscious state (MCS) (1, 2). Advances in intensive care and neurosurgical interventions have led to an increase in the number of patients with DoC, thereby emphasizing the clinical importance of addressing the unique challenges these patients present (3, 4). One of the most significant challenges in managing patients with DoC is the accurate assessment and management of pain, as these individuals are often unable to reliably communicate their suffering (5, 6).

Traditional bedside methods for assessing consciousness have shown a high rate of error, leading to misinterpretations of behavioral signals, such as grimacing, agitation, or changes in muscle tone. Such misjudgments can have profound ethical, clinical, and legal consequences, influencing decisions related to prognosis, treatment plans (6, 7). A critical aspect of managing pain in patients with DoC is the ability to distinguish between reflexive responses and intentional behaviors, however, due to the lack of objective and reliable methods, detecting pain in non-communicative patients remains an ongoing challenge (8).

Current neuroimaging techniques, including functional magnetic resonance imaging (fMRI), electroencephalography (EEG), and positron emission tomography (PET), have been explored to assess pain processing in patients with disorders of consciousness (DoC) (5, 9). However, these methods have notable limitations. fMRI, for example, requires patients to remain still during scans, a task that is often not feasible for patients with severe brain injuries. Furthermore, fMRI is costly and technically complex, limiting its applicability in routine clinical settings. Similarly, while EEG is useful for monitoring brain activity, it often reveals non-specific low-frequency patterns in patients with DoC, complicating the interpretation of pain-related brain responses. PET, although capable of identifying brain activity at a metabolic level, has its own limitations, including the need for radiation exposure and its high cost, which restricts its use in clinical practice. These limitations underscore the need for alternative, more accessible methods to assess pain in patients with DoC (10, 11).

In this regard, near-infrared spectroscopy (NIRS) has emerged as a promising solution (12–14). NIRS is a non-invasive, portable, and relatively cost-effective technology that measures changes in cerebral blood flow, offering real-time insights into brain activity (15). Unlike fMRI, NIRS does not require patient cooperation and can be performed directly at the bedside, making it particularly suitable for patients with DoC (16). NIRS can detect dynamic changes in brain oxygenation and blood flow in response to noxious stimuli, providing valuable information about the brain's response to pain (9).

Although the application of NIRS in patients with DoC is still in the exploratory stage (17), preliminary studies suggest that NIRS can reflect the neural response of patients to pain and may provide an effective method for clinical pain assessment (18, 19). However, research on the use of NIRS for pain evaluation in patients with DoC remains limited, and there is a lack of systematic studies on the impact of nociceptive pain stimuli on brain activity (17).

Therefore, in this study, we applied pressure-induced pain stimuli to patients with DoC and simultaneously collected NIRS data. Pressure-induced pain, a type of mechanical pain caused by external compression, is commonly experienced by patients with DoC due to prolonged immobility, medical devices, and pressure sores (20). Given its clinical relevance, pressure stimulation was selected as the pain-inducing method to better reflect real-world conditions. The aim was to identify changes in brain activity associated with pain perception, provide insights into how the brain responds to noxious stimuli in these non-communicative patients, and lay a solid foundation for the use of NIRS in the clinical management of pain for patients with DoC.

## 2 Materials and methods

### 2.1 Participants

In this study, 15 patients (12 males and 3 females) were recruited from Beijing Tiantan Hospital, Capital Medical University, Civil Aviation General Hospital and Hangzhou Mingzhou Brain Rehabilitation Hospital. Inclusion criteria: (1) various types of brain injuries leading to DoC, including traumatic brain injury (TBI), stroke, hypoxic-ischemic coma (HIE), and meningoencephalitis, etc, with a duration of more than 28 days and in a stable condition. (2) diagnosed as UWS or MCS using the coma-recovery scale-revised (CRS-R) scale. Exclusion: (1) long-term use of sedative or antiepileptic drugs, (2) uncontrollable infections or other serious medical diseases, (3) inability to obtain informed consent from the legal caregivers. In this study, written informed consent for each subject was obtained from the patient's legal guardians. The experimental protocol of this study was approved by the ethics committee of Beijing Tiantan Hospital, Capital Medical University. The clinical characteristics of the patients with DoC are shown in Table 1.

**Table 1:** Clinical characteristics of patients with disorders of consciousness.

**No**.	**Diagnosis**	**Age (years)**	**Gender**	**Duration of DoC (months)**	**Etiology**	**CRS-R score**
1	MCS	59	M	192	HIE	9 (213,102)
2	MCS	44	M	24	Stroke	11 (233,102)
3	MCS	67	M	36	Stroke	11 (331,112)
4	UWS	47	M	6	TBI	6 (103,101)
5	UWS	61	M	31	Stroke	7 (212,002)
6	MCS	32	F	13	TBI	15 (234,213)
7	MCS	64	M	8	Stroke	11 (313,013)
8	MCS	51	M	38	TBI	14 (433,202)
9	UWS	21	M	5	TBI	5 (102,002)
10	UWS	37	F	17	Stroke	4 (002,101)
11	UWS	61	M	21	Stroke	6 (112,002)
12	MCS	35	M	13	TBI	10 (133,102)
13	UWS	65	F	4	Stroke	5 (003,002)
14	MCS	34	M	2	Stroke	13 (234,112)
15	UWS	22	M	11	TBI	6 (112,002)

CRS-R, coma recovery scale-revised; DoC, disorders of consciousness; MCS, minimally conscious state; UWS, unresponsive wakefulness syndrome; M, male; F, female; TBI, traumatic brain injury; HIE, hypoxic-ischemic coma.

### 2.2 Study design

This study employed NIRS to systematically investigate the spatiotemporal characteristics of brain responses to nociceptive stimuli in patients with DoC. The experimental paradigm consisted of an initial resting period (30 s), followed by sequential nociceptive stimulation applied to the left upper limb, right upper limb, left lower limb, and right lower limb. Each stimulation block included a 30-s stimulation period and a 30-s rest period. Nociceptive stimuli were delivered using an electronic algometer targeting the fingernail bed of the middle finger and the toenail bed of the third toe. Pressure was gradually increased until a clear behavioral response (e.g., facial expression changes, limb reflexes, or vocalizations) was observed or the safety threshold of 120 N/cm^2^ was reached. To ensure the efficacy of stimulation, each application lasted no <5 s, with repeated stimulations performed within the 30-s stimulation period. Auditory cues, such as “start stimulation” and “relax” were presented in a pseudo-randomized order to guide participants through the tasks and minimize fatigue or attentional fluctuations that might impact the experimental outcomes. The experimental process was concealed from the MCS patients during the experiment to ensure that their subjective consciousness does not affect the experiment.

### 2.3 Data acquisition

**Figure 1:**
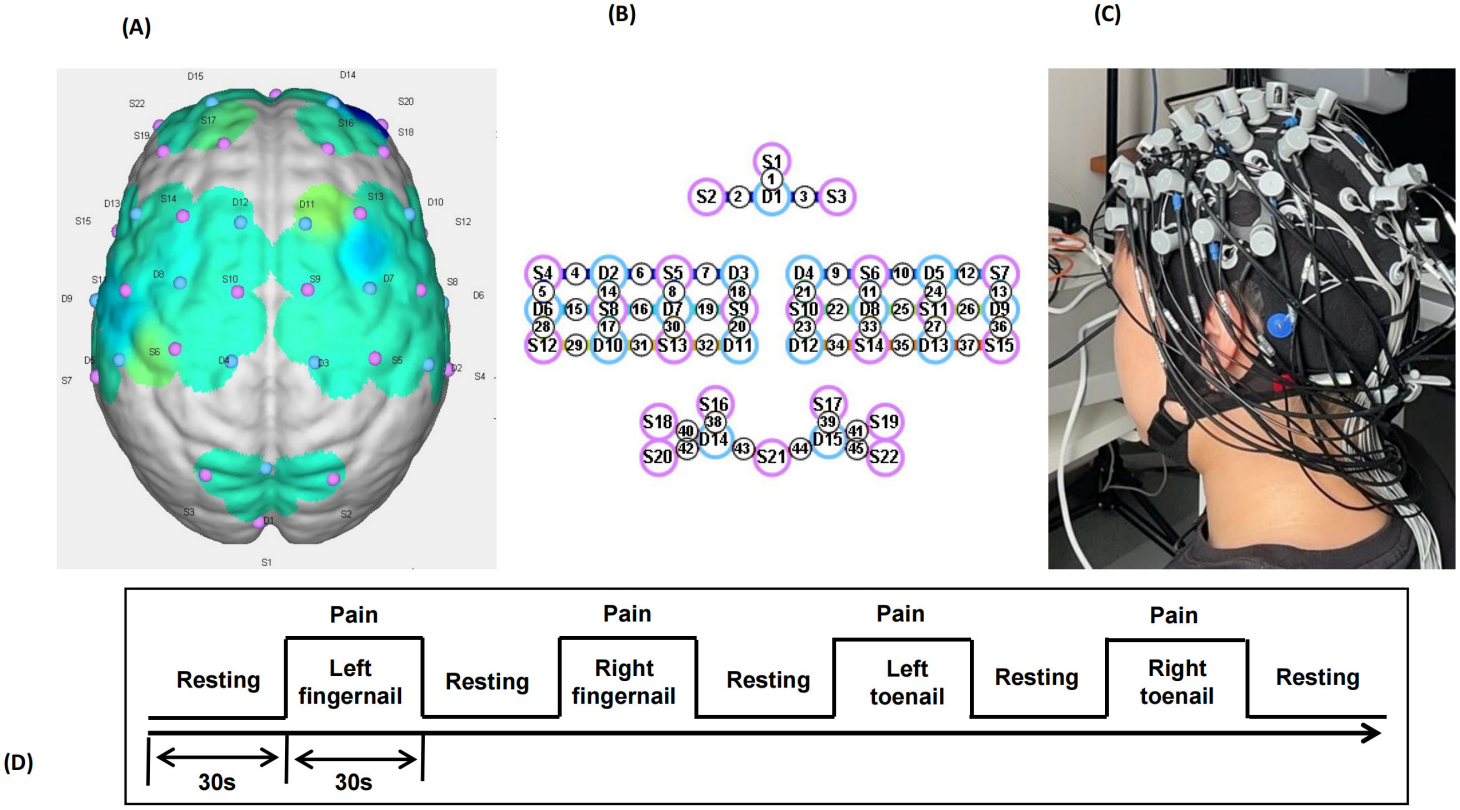
llustration of the experimental configuration. **(A)** Three-dimensional diagram of the arrangement of the optodes. **(B)** Diagram of the arrangement of the optodes. Specifically, we used 22 sources (red circles) and 15 detectors (blue circles), for a total of 45 optical channels. **(C)** Photograph of the experimental setup. **(D)** Photograph of the experimental setup.

NIRS data were acquired using the NirSmart-6000A equipment (Danyang Huichuang Medical Equipment Co., Ltd., China). Two wavelengths, 730 and 850 nm, were used to detect the concentration changes in Oxyhemoglobin (HbO), Deoxyhemoglobin (HbR), and Total Hemoglobin (HbT) of the brain in real-time. The NIRS system consisted of 22 sources and 15 detectors, totally yielding 45 optical channels, the average distance between the source and the detector is 3 cm (range 2.7–3.3 cm), with reference to the international 10/20 system for positioning. The location information of the 45 channels is shown in Figure 1. The red and blue circles represent the light sources and detectors, respectively, while the gray connecting lines marked with numbers indicate the optical channels. The sampling rate of the fNIRS system was 11 Hz.

### 2.4 Data analysis

NIRS data were processed using MATLAB 2019a (MathWorks Inc., Natick, Massachusetts, USA). Raw light intensity data were converted into relative changes in HbO and HbR concentrations using the modified Beer-Lambert law. To ensure data quality, the signal-to-noise ratio (SNR) of each optical channel was calculated using the coefficient of variation (CV = σ/μ), where μ and σ represent the mean and standard deviation of the signal, respectively. Channels with a CV > 15% were excluded, while those with a CV < 5% were retained for further analysis (21).

The data were transformed into optical density, and concentration changes were calculated using molar extinction coefficients for HbO and HbR. A band-pass filter (0.01–0.2 Hz) was applied to remove task-unrelated noise such as heartbeat, breathing, and blood pressure fluctuations. Motion artifacts were identified and corrected using principal component analysis (PCA), with data containing large motion artifacts discarded. In this study, the pain-related regions of interest (ROIs) and their corresponding channels were selected based on previous studies and Brodmann's areas (17, 22). These include the somatosensory association cortex (SAC) (Channels 1, 2, 3), primary somatosensory cortex (PSC) (Channels 4, 6, 7, 8, 9, 10, 12, 13, 14, 15, 24, 26), temporal gyrus (TG) (Channels 4, 5, 13, 28, 36), primary motor cortex (PMC) (Channels 7, 8, 9, 10, 11, 14, 15, 16, 17, 18, 21, 24, 25, 26, 27), frontopolar area (FPA) (Channels 38, 39, 40, 41, 42, 43, 44, 45), and dorsolateral prefrontal cortex (dPFC) (Channels 17, 27, 30, 31, 33, 35, 38, 39, 40, 41, 42, 45). The dataset was divided into three phases: pre-stimulation, during stimulation, and post-stimulation, each lasting 30 s. For each phase, the mean values of the hemodynamic responses (HbO and HbR) were calculated for each ROI, including SAC, PSC, TG, PMC, FPA, and dPFC.

Functional connectivity was assessed by calculating pearson correlation coefficients between the time series of all pairs of optical channels, resulting in a 45 × 45 correlation matrix for each participant. The correlation matrices were visualized across the three phases (pre-stimulation, during stimulation, and post-stimulation) using the BrainNet Viewer toolbox (https://www.nitrc.org/projects/bnv/). For quantitative analysis, the 45 channels were categorized into five brain regions: SAC, PSC, TG, PMC, dPFC. The mean correlation values within each region were extracted for comparisons across different phases.

### 2.5 Statistical analysis

Paired samples *t*-tests were conducted to compare the hemodynamic responses (HRO) and functional connectivity values across the three experimental phases (pre-stimulation, during stimulation, and post-stimulation). The false discovery rate (FDR) correction was applied to account for multiple comparisons and ensure the reliability of the results.

## 3 Results

In this study, we analyzed the HRO and functional connectivity in various ROIs to assess the impact of pain stimulation in patient with DoC.

### 3.1 HRO results

#### 3.1.1 HbO results

**Figure 2:**
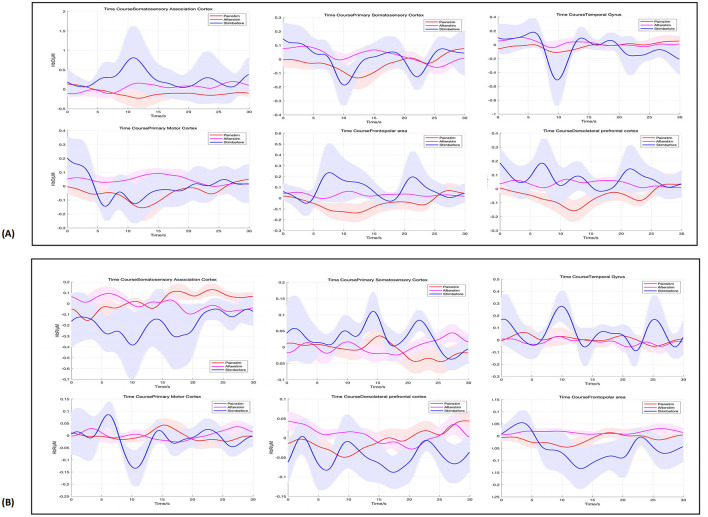
Hemodynamic responses across ROIs, in pre-stimulation (blue), stimulation (red), and post-stimulation (pink) phases. From left to right, are SAC, PSC, TG, PMC, FPA, and dPFC. **(A)** Shows the HbO results, while **(B)** displays the HbR results.

For the comparison between stimulation and baseline, no significant changes in HbO were observed across ROIs. Specifically, the SAC showed a mean change of 0.26 ± 1.83, with a *t*-value of −0.74 and a *p*-value of 0.58, indicating no significant difference. Similarly, the PSC, TG, and PMC exhibited small mean changes (0.01 ± 0.17, −0.07 ± 0.21, and −0.07 ± 0.32, respectively) with corresponding non-significant *t*-values and *p*-values (*t*-values ranging from −0.86 to −0.50, and *p*-values ranging from 0.30 to 0.68). Both FPA and dPFC also showed no significant activation compared to baseline (mean changes of 0.09 ± 0.42 and 0.09 ± 0.33, respectively, with *t*-values of −1.13 and −1.41, and *p*-values of 0.55 and 0.39). For the comparison between stimulation and post-stimulation, again, no significant differences were found. The SAC, PSC, TG, PMC, FPA, and dPFC showed minimal changes in HbO, with mean values of 0.03 ± 0.65, 0.03 ± 0.12, 0.02 ± 0.20, 0.04 ± 0.18, 0.03 ± 0.19, and 0.03 ± 0.15, respectively (Figure 2). The *t*-values ranged from −0.83 to −2.33, and the *p*-values ranged from 0.30 to 0.58, all indicating non-significant changes. The results of paired *t*-tests comparing the stimulation phase to baseline and post-stimulation are summarized in Table 2.

**Table 2:** Statistical analysis of HbO in various ROIs.

**ROIs**	**Stimulation vs. baseline (HbO)**			**Stimulation vs. post-stimulation (HbO)**		
	**Mean** ±**SD**	***t*** **-value**	**FDR-corrected** ***p*****-value**	**Mean** ±**SD**	***t*** **-value**	**FDR-corrected** ***p*****-value**
SAC	0.26 ± 1.83	−0.74	0.58	0.03 ± 0.65	−0.83	0.58
PSC	0.01 ± 0.17	−0.86	0.58	0.03 ± 0.12	−1.83	0.30
TG	−0.07 ± 0.21	0.68	0.58	0.02 ± 0.20	−0.70	0.58
PMC	−0.07 ± 0.32	−0.50	0.68	0.04 ± 0.18	−1.97	0.30
FPA	0.09 ± 0.42	−1.13	0.55	0.03 ± 0.19	−1.55	0.35
dPFC	0.09 ± 0.33	−1.41	0.39	0.03 ± 0.15	−2.33	0.30

ROIs, regions of interest; SAC, somatosensory association cortex; PSC, primary somatosensory cortex; TG, temporal gyrus; PMC, primary motor cortex; FPA, frontopolar area; dPFC, dorsolateral prefrontal cortex; HbO, oxygenated hemoglobin.

#### 3.1.2 HbR results

For the comparison between stimulation and baseline, the HbR results showed no significant changes across the ROIs. The *p*-values for the ROIs were as follows: 0.58 for SAC, 0.66 for PSC, 0.68 for TG, 0.80 for PMC, 0.58 for FPA, 0.75 for dPFC, and 0.80 for all regions, indicating a lack of significant activation in these areas. Similarly, for the comparison between stimulation and post-stimulation, no significant differences were observed in the HbR measures. The *p*-values were 0.62 for SAC, 0.75 for PSC, 0.58 for TG, 0.80 for PMC, 0.58 for FPA, 0.72 for dPFC, and 0.80 for all regions, further confirming that no significant activation occurred in these areas post-stimulation (Figure 2).

These findings suggest that while pain stimulation induced minimal changes in brain activity in the selected ROIs, no significant differences were observed in either the stimulation vs. baseline or stimulation vs. post-stimulation comparisons. The lack of significant activation could indicate that pain perception and processing may be impaired or less responsive in patients with DoC, requiring further investigation.

### 3.2 Functional connectivity across ROIs

**Figure 3:**
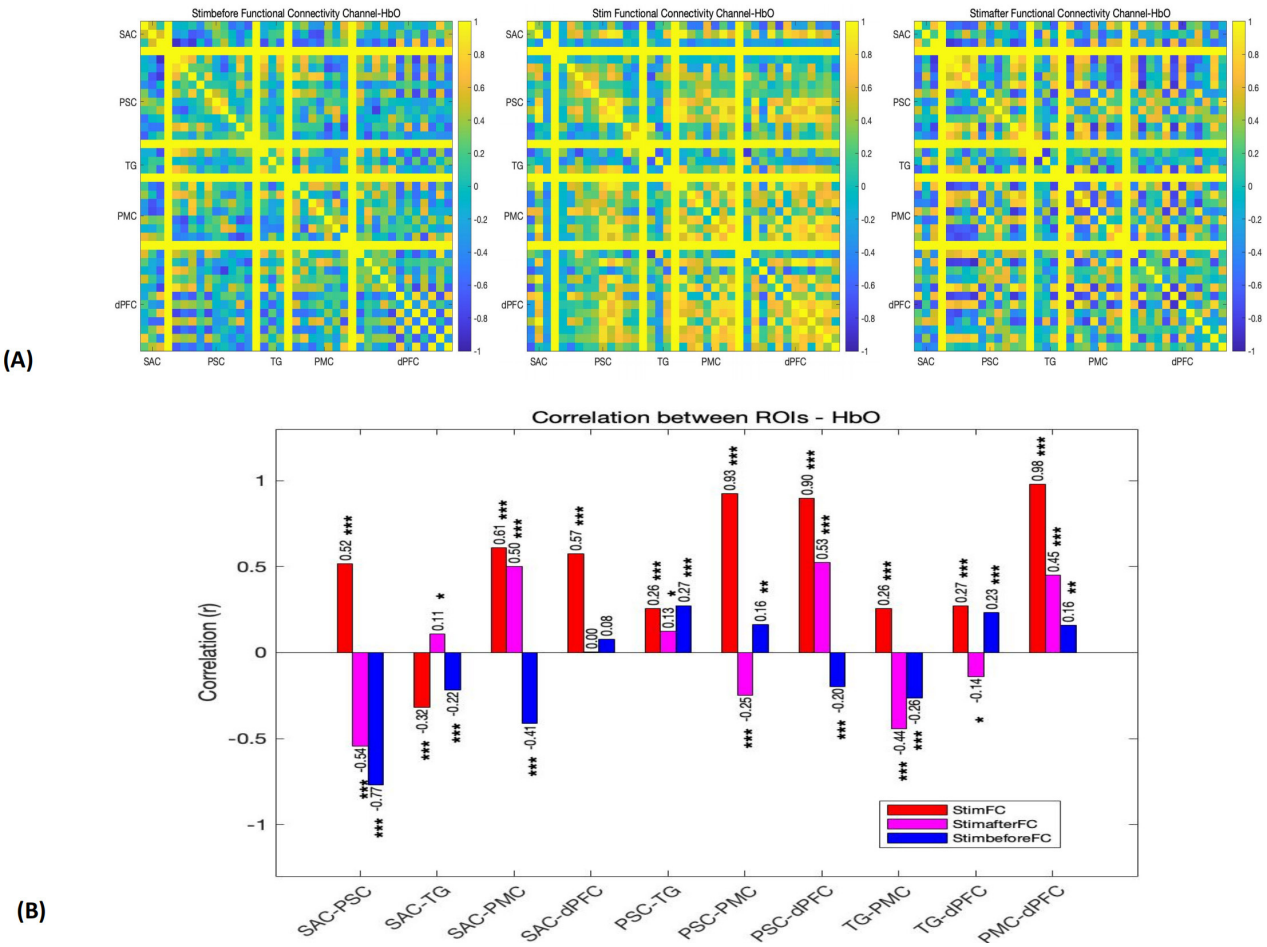
**(A)** Functional connectivity matrices depicting the connectivity patterns for each channel in pre-stimulation, stimulation, and post-stimulation phases (*r*). **(B)** Functional connection coefficient between any two ROIs, in pre-stimulation (blue), stimulation (red), and post-stimulation (pink) phases. *p < 0.05. **p < 0.01. ***p < 0.001.

Then, we assessed functional connectivity across ROIs using cortical HbO levels in patients with DoC during pre-stimulation, stimulation, and post-stimulation phases. The results revealed that the functional connectivity during the stimulation phase was significantly higher than during the pre-stimulation and post-stimulation phases (Figure 3).

#### 3.2.1 Pre-stimulation phase

Before the stimulation, the functional connectivity between most regions was relatively low. Notably, a significant negative correlation was observed between SAC and PSC (*r* = −0.77), suggesting an inverse relationship between these two regions. Additionally, there was a weak positive correlation between SAC and dPFC (*r* = 0.08), and weak connectivity between PMC and other regions (*r* < 0.5). These findings indicate that, prior to the pain stimulation, the brain's network activity was somewhat dispersed, with minimal inter-regional coordination.

#### 3.2.2 Stimulation phase

During the stimulation phase, a significant enhancement in functional connectivity was observed, particularly between regions involved in sensory, motor, and cognitive functions. Strong positive correlations were found between PSC and dPFC (*r* = 0.93), PSC and PMC (*r* = 0.93), and dPFC and PMC (*r* = 0.98), all with *p*-values < 0.001. These high correlations indicate a high degree of coordination between sensory and motor regions, as well as between the motor cortex and the dorsolateral prefrontal cortex, likely reflecting the interplay between pain-induced motor responses and cognitive regulation. In addition, SAC demonstrated moderate connectivity with PSC, dPFC, and PMC, with *r*-values above 0.5. However, TG showed weaker connectivity with other regions, particularly with SAC (*r* = −0.32) and PSC (*r* = 0.26).

#### 3.2.3 Post-stimulation phase

In the 30 s following the stimulation, the functional connectivity remained relatively strong between SAC and PMC (*r* = 0.50) and between PSC and dPFC (*r* = 0.53), though these correlations were notably weaker compared to the stimulation phase. Furthermore, SAC and PSC exhibited a return to negative correlation (*r* = −0.54), indicating a shift in network dynamics after the stimulus was removed.

## 4 Discussion

In this study, we applied pressure stimulation to induce pain and used NIRS to analyze brain activity and functional connectivity by measuring changes in HbO concentrations within the ROIs. While we did not observe significant activation in the ROIs, we found substantial changes in the functional connectivity between key brain areas, including the PSC, PMC, and dPFC. Notably, the correlation coefficients between these regions exceeded 0.9, suggesting that NIRS has the potential to identify pressure-induced pain in patients with DoC, with functional connectivity of HbO may serving as a sensitive indicator for assessing pain in patients with DoC.

### 4.1 Rationale for pressure stimulation and ROI selection

Patients with DoC face numerous potential sources of pain, common causes include fractures, solid organ injuries, soft tissue injuries, and the use of medical devices such as tracheal tubes, nasogastric tubes, and urinary catheters, prolonged immobility often leads to additional complications, such as skin breakdown or pressure sores and soft tissue contractures (23). Multiple studies have confirmed that patients with DoC retain residual pain perception (24–26). Timely and accurate identification of pain not only aids in optimizing individualized treatment plans but also effectively alleviates patient suffering and improves their quality of life. Furthermore, different types and intensities of pain may involve distinct neural mechanisms (5). Thus, precise pain identification is critical for comprehensively understanding the diversity of pain and provides a scientific basis for developing more effective interventions.

Previous pain studies have predominantly focused on electrical and thermal stimulation, which are widely used in experimental research due to their ability to rapidly and intensely induce pain responses (26, 27). However, these stimulation methods may differ mechanistically from real-world clinical scenarios. In contrast, pressure-induced pain, a type of mechanical pain caused by external physical pressure or compression, is a common source of discomfort for patients with DoC in clinical environments (20). Therefore, to better align with the pain situations faced by patients with DoC in real-world clinical contexts and to broaden the scope of pain research, this study adopted pressure stimulation as the pain-inducing method.

The pain neuromatrix in the intact central nervous system is complex and not fully understood. Previous studies have reported that regions such as the anterior cingulate cortex, dorsal horn, insular cortex, periaqueductal gray, prefrontal cortex, rostral ventral medulla, primary somatosensory cortex, and secondary somatosensory cortex are involved in pain transmission and processing, particularly in processing the qualitative aspects of pain, including its localization, intensity, and duration (28–30). Due to the limitations of NIRS, particularly in detecting deeper subcortical regions, we focused on cortical areas in this study. We selected regions that are closely associated with pain perception, emotional regulation, and cognitive processing, including the SAC, PSC, TG, PMC, FPA, and dPFC. Specifically, SAC and PSC are central to the early stages of pain perception, where sensory information is integrated and pain localization occurs (31). TG, including structures such as the hippocampus and amygdala, is involved in the emotional evaluation and processing of pain, but these structures are deep brain structure that lies beyond the penetration depth of fNIRS, making it inaccessible for direct measurement (32). PMC plays a key role in the motor responses associated with pain (33). Lastly, FPA and dPFC are essential for cognitive regulation and emotional inhibition of pain, facilitating emotional adjustment and decision-making in response to pain (28, 29).

### 4.2 Limited brain activation in response to pressure stimulation in patients with DoC

In this study, we did not observe significant changes in HbO and HbR in the ROIs during either the stimulation vs. baseline or stimulation vs. post-stimulation comparisons, despite the application of strong pressure stimulation. Several factors may explain the limited activation of pain-related sensory areas. First, pain perception in patients with DoC remains a controversial issue, although there is growing evidence of residual pain experience. Brain damage in patients with DoC may impair their ability to perceive pain. Previous fMRI, PET and EEG studies have shown that pain can only partially activate primary pain perception areas in the brain, with reduced activation of the entire “pain brain matrix” especially in UWS patients (34, 35). This suggests that the loss of integrated pain processing within cortical and subcortical networks may restrict the brain's ability to fully respond to pain stimuli. Second, the nature of the pain stimulus itself may also contribute to the lack of significant changes. Electrical stimulation typically produces rapid and intense pain responses by directly activating neural pathways, whereas pressure-induced pain, a form of dull mechanical pain, induces a slower and more complex physiological reaction. Pressure pain may not effectively engage higher-level pain processing circuits in the brain, and its lower intensity compared to electrical stimulation could explain the absence of significant brain activity changes (7, 20, 26). Finally, chronic pain or prolonged exposure to painful stimuli in patients with DoC may lead to adaptive changes in the brain's pain processing system. Long-term exposure to pain can enhance pain tolerance or suppress pain perception, leading to a reduced neural response to new pain stimuli (17). Consequently, even when a sufficient pain threshold is reached, the brain's reaction to pressure stimulation remains relatively weak.

### 4.3 Enhanced functional connectivity in response to pressure stimulation in patients with DoC

To further investigate the impact of pain stimulation on the coordinated activity of brain regions, we conducted an analysis of the functional connectivity between ROIs (36, 37). These measures help reveal how different brain regions communicate and coordinate to process sensory and emotional information, providing a more holistic understanding of pain perception and response in patients with DoC (38, 39). Although pressure stimulation did not induce a significant activation in the ROIs, we observed significant changes in functional connectivity between different ROIs under pressure pain stimulation. Specifically, the connectivity between PSC and PMC, PMC and dPFC, as well as PSC and dPFC, showed correlation coefficients exceeding 0.9, with all comparisons yielding *p* < 0.001. Moreover, the gradual restoration of connectivity following stimulation indicates that the brain adjusts its functional network in response to the pain stimulus. This finding suggesting despite the potential impairment in pain perception in patients with DoC, the brain can still integrate information through functional coordination between specific regions. Functional connectivity may help adjust the brain's overall response to pain, potentially linked to emotional evaluation, motor responses, and cognitive processing of pain. Specifically, the connection between the PSC and PMC may indicate that, even without direct pain perception, the brain regulates its overall response to pain through motor preparation and emotional evaluation (29, 33).Furthermore, the enhanced connectivity of dPFC, a region closely associated with cognitive control and emotional regulation, may reflect the brain's mechanisms for emotional suppression and decision-making adjustment when processing pain experiences (29). Despite a potential reduction in pain perception, the brain continues to regulate the emotional and behavioral responses to pain through these higher cognitive and emotional processing network.

This study aims to explore potential biological markers of acute pressure pain in patients with DoC using NIRS. Enhanced functional connectivity in response to pressure stimulation in patients with DoC suggest that NIRS has the potential to identify pressure induced pain in patients with DoC, with functional connectivity of HbO may serving as a more sensitive indicator for assessing pain than activation of pain-related brain areas in patients with DoC. Given that biological markers are characterized by their generalizability (5), our experimental design did not specifically distinguish between patients in UWS and MCS, as these two groups share common challenges such as brain dysfunction and an inability to express pain effectively. Their clinical presentations often overlap, sometimes making it difficult to differentiate between them in clinical practice. To enhance the broader applicability of our findings, we focused on developing pain biomarkers that can be applied to all patients with DoC, rather than restricting the research to specific diagnostic categories.

### 4.4 Limitations

We acknowledge that our study has certain limitations. First, the small sample size (*n* = 15) may impact the generalizability and statistical power of our findings. However, recruiting patients with DoC is inherently challenging due to both practical and ethical constraints (39). Additionally, pain stimulation, as a noxious stimulus, presents ethical and psychological challenges when applied to patients with DoC. In this study, due to the severity of the patients' conditions and the hesitance of their families to accept prolonged pain stimulation, only a single test session was conducted. While this approach provides preliminary data and an initial assessment of pain perception, the limitation of a single test session may not fully reflect the neural responses and adaptive changes in patients under sustained stimulation. Moreover, the presence of structural brain damage among the patients may have influenced our findings. Given the complexity of the patient population and the potential variability in pain intensity and response due to individual differences, future studies could optimize experimental design by incorporating more diverse and repeated stimulation sessions to enhance the reliability and representativeness of the data. Considering the familial concerns regarding prolonged pain stimulation, future research could explore non-invasive pain induction methods or a gradual stimulation approach, which would allow for further exploration of pain-related brain responses without increasing patient distress.

## 5 Conclusion

This study investigated the effects of pressure-induced pain on brain activity in patients with DoC using NIRS. Although no significant activation was observed in the ROIs, we found notable changes in functional connectivity between key pain-related regions, such as the PSC, PMC, and dPFC. These changes suggest that, despite impairments in pain perception, the brain continues to process pain information through coordinated activity across multiple regions. Our findings highlight the potential of functional connectivity as a more sensitive measure of pain processing in patients with DoC based on NIRS.

## Data Availability

The datasets presented in this study can be found in online repositories. The names of the repository/repositories and accession number(s) can be found in the article/supplementary material.
